# Using a Floating-Gate MOS Transistor as a Transducer in a MEMS Gas Sensing System

**DOI:** 10.3390/s101110413

**Published:** 2010-11-18

**Authors:** Mario Alfredo Reyes Barranca, Salvador Mendoza-Acevedo, Luis M. Flores-Nava, Alejandro Avila-García, E. N. Vazquez-Acosta, José Antonio Moreno-Cadenas, Gaspar Casados-Cruz

**Affiliations:** CINVESTAV-IPN, Electrical Engineering Department, Av. IPN No. 2508, Col. San Pedro Zacatenco, Mexico, D.F. 07360, Mexico; E-Mails: smendoza@cinvestav.mx (S.M.-A.); lmflores@cinvestav.mx (L.M.F.-N.); aavila@cinvestav.mx (A.A.-G.); neo_wolfx@hotmail.com (E.N.V.-A.); jmoreno@cinvestav.mx (J.A.M.C.); gcasados@solar.cinvestav.mx (G.C.-C.)

**Keywords:** floating-gate MOSFET, MEMS, gas sensor

## Abstract

Floating-gate MOS transistors have been widely used in diverse analog and digital applications. One of these is as a charge sensitive device in sensors for pH measurement in solutions or using gates with metals like Pd or Pt for hydrogen sensing. Efforts are being made to monolithically integrate sensors together with controlling and signal processing electronics using standard technologies. This can be achieved with the demonstrated compatibility between available CMOS technology and MEMS technology. In this paper an in-depth analysis is done regarding the reliability of floating-gate MOS transistors when charge produced by a chemical reaction between metallic oxide thin films with either reducing or oxidizing gases is present. These chemical reactions need temperatures around 200 °C or higher to take place, so thermal insulation of the sensing area must be assured for appropriate operation of the electronics at room temperature. The operation principle of the proposal here presented is confirmed by connecting the gate of a conventional MOS transistor in series with a Fe_2_O_3_ layer. It is shown that an electrochemical potential is present on the ferrite layer when reacting with propane.

## 1. Introduction

Since the past decade, efforts are still ongoing to improve issues like performance, configuration, fabrication, and cost yields for gas sensors. Factors like low power consumption, small size, monolithic integration of the sensor stage together with the controlling and conditioning electronics, and batch fabrication are contributing to achieving these targets, but the mechanism for gas sensing can also be considered an important feature of the integrated system that can influence the kind of sensor to be used or fabricated. In particular, semiconductor gas sensors (SGS) are based on the following classification: (a) *chemiresistor sensors*, (b) *chemicapacitor sensors*, (c) *chemimechanical sensors*, (d) *electrochemical sensors*, and (e) *optical sensors* [1–3]. Although all of these gas sensor principles can be achieved using available standard CMOS technology, there are specific post-processing steps for each one that determine system operation issues such as sensitivity range, operating temperature, species detected, associated electronics, *etc.* Among the simplest but still reliable sensors, we can mention a gas sensor that is included within the electrochemical classification, relying on charge transfer reactions. In turn, electrochemical sensors can also be classified into the following [1]: (1) *voltammetric sensors*, related with the measurement based on a current-voltage relationship, (2) *potentiometric sensors*, if a potential is measured with no current flowing, or (3) *conductometric sensors*, when the measured variable is the conductance of a film between two electrodes. As mentioned above, one of the most important objectives is to integrate on the same substrate either the sensor or the actuator together with the required control and signal processing circuitry. This fact has a high impact on the end product since it represents a reduction of typical problems present in hybrid systems such as parasitic capacitances and crosstalk among electronic circuits. Hence, different design approaches using analog, digital or mixed signal electronic systems can be implemented to configure a complete semiconductor gas sensor. Finally, costs can be reduced since a monolithic integration can be achieved with compatible CMOS technology allowing for batch production of chips having a low cost mass production [4–11].

On the other hand, semiconductor gas sensors use a variety of semiconducting metal oxides as the sensitive layer. These layers are deposited between two metallic electrodes from where a change in resistance is measured while they are in contact with a gas. Commonly, metal oxides like SnO_2_, ZnO, WO_3_ and Fe_2_O_3_ are used to detect oxidizing or reducing gases but organic polymers can also be used for detection of organic compounds. When metal oxides are used, they must be locally heated. This can be achieved by using a polysilicon micro-heater [12–14]. Heat then promotes a chemical reaction between the metal oxide and the gaseous species. Usually the temperature range is 200 °C–600 °C. The interaction between oxygen adsorbed at the layer surface and the gas in the environment, changes the population of surface electrons or holes. Although this change in carrier population certainly produces a change of resistance of the sensitive layer relative to the gas concentration, the charge exchange can be handled instead. Hence, an alternative for gas sensors can be based on MOS transistors since they are sensitive to charge present either on the gate or within the channel oxide. Therefore, the threshold voltage shift can be correlated with gas concentration.

This paper presents a proposal of an integrated gas sensor that can be fabricated with standard CMOS technology. The only extra steps added after the chip fabrication, are a post-processing anisotropic etching for bulk micromachining a thin membrane where the micro-heater is located, and the deposition of the sensitive layer. In this way, the circuitry can operate at room temperature while the micro-heater reaches temperatures above 200 °C, both on the same silicon substrate. Therefore compatibility of CMOS fabrication with MEMS technology gives also a MEMS category to the SGS. Design considerations for the mentioned micro-heater include small size, low power consumption and mechanical robustness to support high temperatures keeping its integrity. Accordingly, electro-thermal coupling among the different elements used in the system must be optimized for a proper operation of the semiconductor gas sensor. In addition, the working principle of the sensor based on the floating gate MOSFET (FGMOSFET) used as the transducer, is explained. Also, the reliability for gas sensing is demonstrated, provided that it can be easily integrated with standard CMOS technologies that offer at least two polysilicon layers and two metals.

## 2. Experimental Description

### 2.1. Gas sensing with FGMOSFET

**Figure 1.**
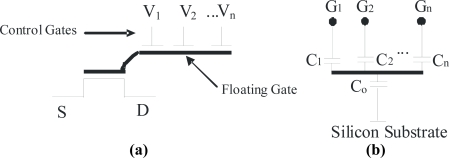
**(a)** Symbol of a multi-input gate FGMOSFET; **(b)** Capacitive equivalent circuit.

MOSFETs are nowadays the most popular devices used for modern integrated circuit systems. The fabrication technology is very mature and advantage can be taken of the properties of such a charge sensitive device. Sensor designs have been reported using a so called floating gate made by standard technologies, but they are used for pH measurement of liquid solutions [15–17] without using two polysilicion layers. A hydrogen sensor using metals such as Pt, Ir or Pd for the gate of the transistor was also reported [18,19]. Such metals are not commonly available as MOS gates in these kinds of technologies. On the other hand, FGMOSFETs (using two polysilicon layers) are widely used as digital memories since they have a long term charge retention that makes them non-volatile devices, conveniently storing the desired information [20–24]. Plenty of literature can be found related to the diverse applications given to FGMOSFETs in items such as neural networks, instrumentation electronics [25,26], soft-hardware logic (SHL), *etc.* [27,28] The name of neu-MOS (ν-MOS) is given when it is designed with one or more control gates since it simulates the operation of a biological neuron [27]. Due to the multiple inputs, the corresponding signals are added at the floating gate and the transistor will be either on or off depending on the sum value. The symbol of this device is shown in Figure 1(a).

It can be seen that the floating gate has no electrical connection since it is completely surrounded with silicon dioxide. Figure 1(b) shows the equivalent circuit of a FGMOSFET multiple input gate, represented by a voltage divider formed with capacitors. From this circuit, it is easily shown that the floating gate voltage is a weighted sum of the voltages applied on the control gates. The floating gate voltage can be found with the following expression:

(1) $$\begin{array}{l} V_{\mathrm{FG}}=\overset{k}{\sum }\frac{C_{k}}{C_{T}}V_{\mathrm{CGk}}+\frac{Q_{\mathrm{FG}}}{C_{T}}\\ \;\;\;\;\;\;\;\;\;\;\;\;\;\;\;C_{T}=\overset{k}{\sum }C_{l}\\ \;\;\;\;\;\;\;\;\;\;\;\;\;\;\;k=1,2,3,\ldots ,n\;\;\;\;\;\;\;l=0,1,2,\ldots ,n \end{array}$$

where *V_FG_* is the floating gate voltage, *C_0_* is the gate capacitance, *C_k_* are the control gate capacitances, *Q_FG_* is the charge present over the floating gate and *n* is the number of control gates of the neu-MOS. So, the floating gate voltage depends both on the voltage applied over the control gates and any charge that could be present at the floating gate.

Two mechanisms are normally used to inject (extract) charge to (from) the floating gate: (a) *hot electron injection* and (b) *carrier tunneling*. Both are based on the electric field established between proper terminals of the devices, causing a current flow through the oxide between the correspondingly biased capacitor plates. Regarding the proposed structure for the SGS, a third mechanism for charge modification can be added to the operation of FGMOSFETs that can be named *chemical charge injection* where no current flow is present. As mentioned before, when reducing or oxidizing gases react with a metallic oxide (or polymers when detecting organic aromas), free electrons are either released to or taken from the surface according to the following chemical reactions, with the help of proper temperatures [12]:

(2) $$O_{2}+2e^{-}\to 2O^{-}$$

(3) $$H_{2}+O^{-}\to H_{2}O+e^{-}$$

(4) $$\text{CO}+O^{-}\to {\text{CO}}_{2}+e^{-}$$

**Figure 2.**
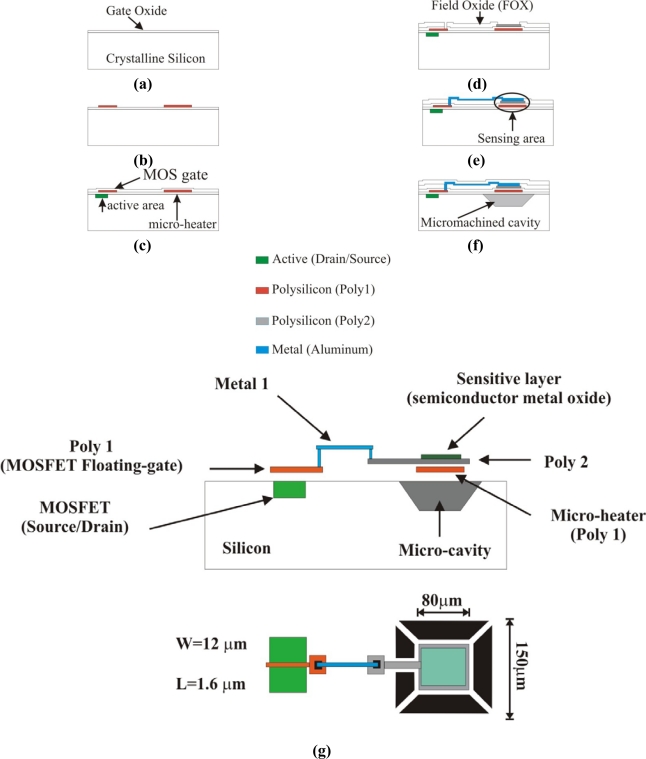
Scheme of the proposal for an integrated gas sensor with FGMOSFET. **(a)** Silicon wafer oxidation; **(b)** Poly1 growth and definition in the MOSFET active area and micro-heater; **(c)** Second oxidation; **(d)** Poly2 growth and definition of the sensing area; contact windows definition; **(e)** Metallization and interconnection definition; **(f)** Micromachining wet post-process for the thermal isolation of the thin membrane; **(g)** Cross section and layout (not to scale) of the suggested gas sensor system.

Then, when an oxygen molecule present in the air is adsorbed on the layer surface, in the presence of a high enough temperature, it dissociates forming O^−^. This process needs two free electrons which are extracted from the layer. If instead hydrogen (or any other reducing gas) enters in contact with these ions, the byproduct is water and an electron is injected back to the layer. The same mechanism is followed when the reaction is carried out with carbon monoxide (or any other reducing gas). The amount of charge is a function of gas concentration, so there can be a corresponding correlation. Usually, this correlation is monitored measuring the resistance variation of a layer placed between two metallic electrodes. However, if this *chemical charge injection* is used in a floating gate, a useful alternative can be conveniently handled through FGMOSFETs. Figure 2 illustrates the suggested configuration for the FGMOSFET to be used as a gas sensor.

By using a standard CMOS technology that offers at least two polysilicon layers and two metals (AMI 1.5 microns, for instance), this structure can be fabricated based on known layout outlines adapted to support MEMS micromachining processes [29,30]. Using this process a membrane supported by four thin arms at each corner can be conveniently obtained. The standard fabrication process can be shortly described following Figure 2. First, an oxidation is made over the silicon substrate surface [Figure 2(a)]. Then a deposit of a first layer of polysilicon (Poly1) is made and defined with photolithography using a first mask, configuring both the MOS gate and the micro-heater [Figure 2(b)]. With the polysilicon gate defined, other windows are opened with a second mask defining the active area of the MOS transistor which allows the diffusion of drain and source terminals. Following, a second oxidation is made over the wafer surface and the Poly1 [Figure 2(c)]. Next, above this last oxide a second layer of polysilicon (Poly2) is grown (Figure 2d) and defined as the sensing area using a third mask. The gas sensitive layer will be deposited over this polysilicon layer after the integrated circuit is fabricated. Again, an oxidation is made over the wafer’s surface. With the help of a forth mask, windows are opened through the silicon dioxide, where metal contacts are required, i.e., Poly2 and the gate (Poly1). Then, aluminum is evaporated and using a fifth mask, interconnections between the sensing area and the MOS gate are defined [Figure 2(e)]. As the following step, a passivation glass is deposited covering the entire surface of the integrated circuit so the integrated circuit is protected against atmosphere contamination. In the last step, windows are opened through the passivation glass and the silicon dioxide, down to the surface of the silicon wafer. These windows are made with a sixth mask and define the areas where an etching post-process should be made leaving a thin membrane at the sensing area [Figure 2(f)]. This step is made following well known anisotropic etching processes by using solutions like tetramethylammonium hydroxide (TMAH). Figure 2(g) shows a cross section together with a layout of the system. Here it should be remarked that Poly1 is normally the layer used as the floating gate when conventional FGMOSFETs are laid out, but with this design a modification has to be done to extend the gate over Poly2 as it can be noticed in Figure 2(g). The gate of the MOS transistor (Poly1) is connected to Poly2 (sensing area) located over the micro-heater via a strip of aluminum (Metal1). Any charge present over Poly2 due to the chemical reaction will be distributed along the node, creating in consequence an equipotential surface with electric influence also in the channel under the gate of the MOS transistor. On the other hand, this Poly2 should be kept protected from the etching solution during the micromachining post-process, otherwise the Poly2 layer will be etched away. This can be achieved since two layers of aluminum (Metal1 and Metal2) are laid out covering the Poly2 sensing area. One or more control gates can be added to the FGMOSFET (not illustrated in Figure 2), depending on the control and signal processing circuits configured for the operation of the system.

### 2.2. Integrated Micro-Heater Features and Design

As mentioned before, heat is needed so the chemical reaction can take place between the metal oxide and reducing or oxidizing gases. The electro-thermal properties of polysilicon can be used for this purpose. If a resistance is made with this material, the Joule effect can be used to heat it up. There are two targets regarding the operation of the micro-heater: (1) a low power consumption within the operating temperature range must be assured, and (2) good thermal isolation between the hot thin membrane after micromachining and the rest of the silicon substrate where the circuitry is placed, must be proven.

#### 2.2.1. Polysilicon Temperature Coefficient of Resistance

**Figure 3.**
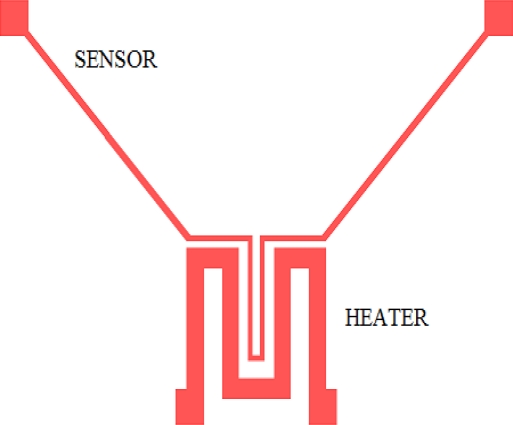
Layout of the micro-heater together with the temperature sensor, made with Poly1.

A useful property of polysilicon for our goals is its *temperature coefficient of resistance* (TCR), *i.e*., the fact that its resistance value increases as its temperature is raised. Hence, by making an electrical current flow along a polysilicon resistor constituting a micro-heater, the metallic oxide thin film can be locally heated. Clearly, the operating temperature must be controlled and limited to avoid overheating and damage of the structure. Therefore, the relation between the polysilicon resistance and its temperature must be known. In turn, this relation can be extrapolated towards calculation of the power consumption of the heater. Based on the results, the geometry of the micro-heater can be adjusted to optimize its operation. First of all, it is suggested to layout the micro-heater in a small area, with a low resistance value. It should fit into the area where the sensing layer is located. Besides, it is convenient to integrate close to the micro-heater a temperature sensor made also with Poly1. By using the same thermal property of polysilicon and the measured TCR, this temperature sensor can measure the actual temperature of the micro-heater, which at the same time is used by a temperature controlling circuit. Figure 3 shows a layout of the micro-heater and the temperature sensor design for the SGS using Poly1.

**Figure 4.**
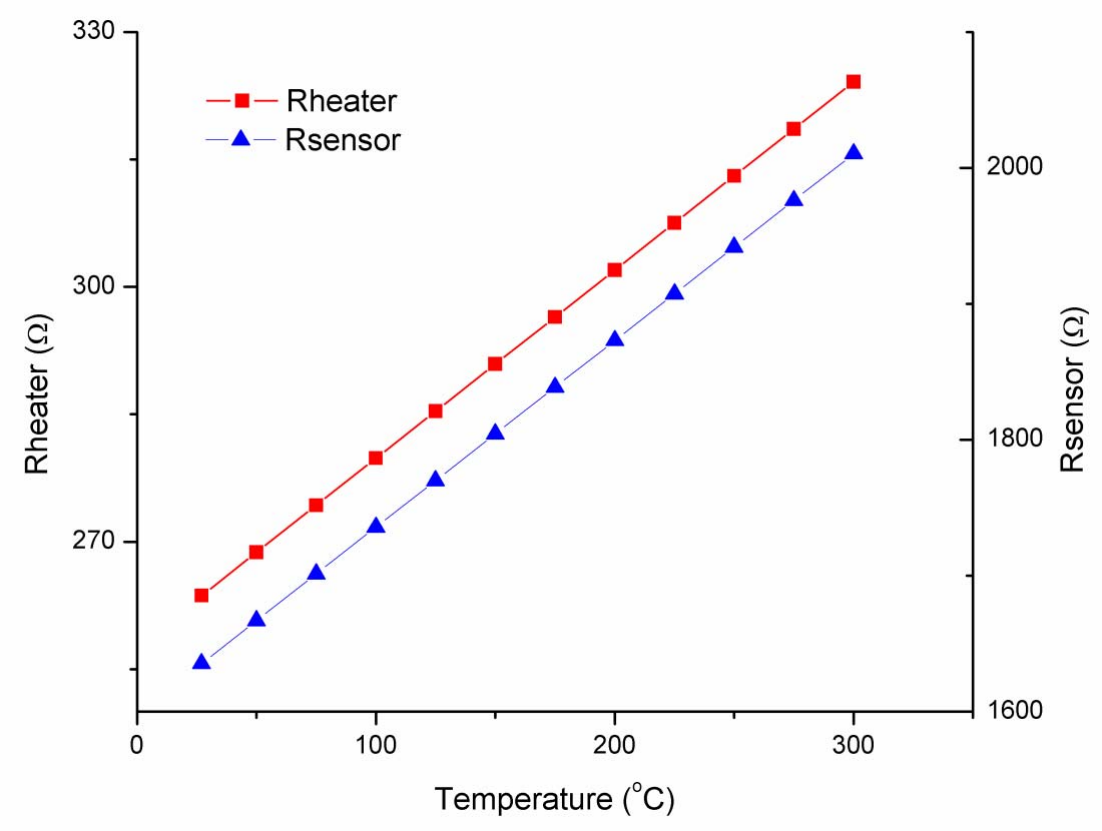
TCR obtained from the multiphysics simulation, for polysilicon micro-heater and temperature sensor.

Sheet resistance of Poly1 from AMI’s 1.5 μm technology has a mean value of 25.1 ohms sq. Then a layout was made for the micro-heater having a total resistance of 263 Ω and a temperature sensor with a resistance of 1.6 kΩ. The polysilicon resistance value is related to temperature by the following expression:

(5) $$\frac{R-R_{0}}{R_{0}}=\mathrm{TCR}(T-T_{0})$$

where *T_0_* is room temperature and *R_0_* is the corresponding resistance value. TCR is a material’s constant and can be experimentally determined, like *R_0_*. The experimentally measured TCR value was 8.84 × 10^−4^ 1/°C. From (5), it is clear that polysilicon can be used as a temperature sensor as well. Regarding the micro-heater, when a voltage is applied across its terminals, the temperature is raised due to Joule effect and in consequence, the polysilicon resistance value increases, as is shown in Figure 4.

**Figure 5.**
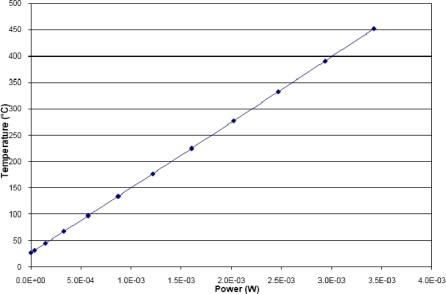
Power consumption for the micro-heater, calculated using the TCR obtained in Figure 4.

In order to find the relationship between temperature and power, the structure was simulated with COMSOL® [31] using an electro-thermal model with proper material data and boundary conditions. Voltages applied to the micro-heater terminals in the simulation were from 0V to 1.1 V. The maximum temperature reached was 452 °C. From this simulation, a TCR value of 8.04 × 10^−4^ 1/°C was obtained, very close to the measured value. Thus, once the TCR is known, the power efficiency can be determined with the plot shown in Figure 5, giving a value of 124 °C/mW. Although this is a value obtained from a simulation, the design suggests a low power consumption operation.

### 2.3. 2D and 3D Electro-Thermal Simulation

Since the temperature sensor is in close proximity with the micro-heater, as shown in the layout of Figure 3, temperature changes are the same in both elements due to heat conduction across the surrounding silicon dioxide. Hence, a thermal coupling should be considered taking into account the characteristics of all the layers used in the structure, *i.e*., the thermal properties of polysilicon, silicon dioxide and the aluminum interconnections. Also, the way the structure is configured establishes the thermal response time for both the micro-heater and the temperature sensor, when heating or cooling.

**Figure 6.**
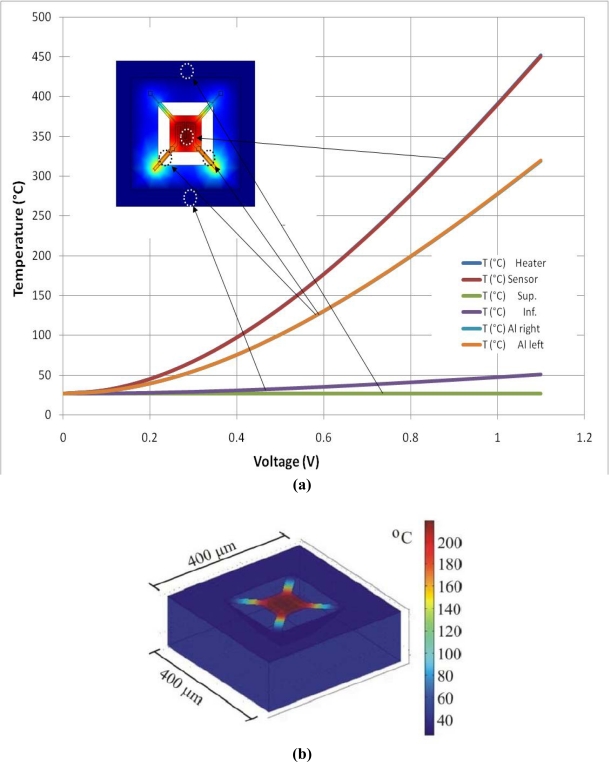
**(a)** 2D micro-heater model. Temperature at six different positions (the inset shows the respective positions over the micro-heater model); **(b)** 3D micro-heater model showing a high temperature concentrated within the membrane.

With this kind of systems, where a high temperature is needed to promote the chemical reaction of the gas with the metallic oxide, concern arises about the operating temperature of the circuitry that is integrated upon the same substrate, since the micro-heater must operate at temperatures around 200 °C while ideally the electronics should operate at room temperature. This is solved with the bulk etching based on MEMS micromachining processes. Again, with the help of COMSOL, a multiphysics software implemented for analysis based on finite element, the temperature distribution over and around the sensing area, can be obtained. 2D and 3D solid models were created, similar to the proposed design and simulated with voltages applied to the micro-heater terminals. Figure 6(a,b) shows the result of the simulations, where the temperature distribution demonstrates that although the temperature is about 200 °C in the micro-heater zone, the surrounding silicon beyond the micromachined region keeps at near room temperature, which confirms the reliability of this structure for temperature isolation.

The 2D model illustrated in Figure 6(a), shows the temperature attained at different zones across the structure. As it can be seen, a voltage sweep from 0 V to 1.1 V was applied to the micro-heater. At the upper and lower edges, the surface reaches 50 °C at the maximum voltage; at the edge of the membrane arms, the temperature is around 325 °C, while at the center of the micro-heater, 450 °C are reached. Figure 6(b) shows a 3D model, where it can be seen that the bulk also keeps at a temperature well below the maximum operating temperature.

**Figure 7.**
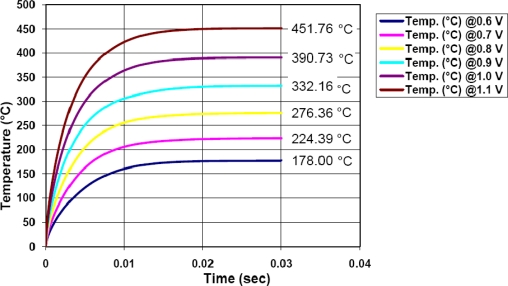
Simulated transient response applying 0.6V, 0.7V, 0.8V, 0.9V, 1.0V and 1.1V to the micro-heater.

Figure 7 shows the transient response of the micro-heater with applied voltage as parameter. It is seen that the micro-heater temperature reaches a steady state after 20 ms approximately. This is a rather good rising time that can assure a fast response of the system. Although the micro-heater region is formed with layers like polysilicon, silicon dioxide, aluminum and passivation glass, finally it is not too massive and is able to quickly heat up or cool down.

**Figure 8.**
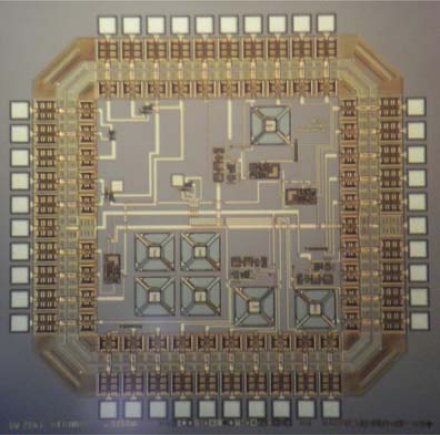
SEM microphotograph of the fabricated prototype of the gas sensor, using FGMOSFETs.

A prototype system was fabricated through MOSIS with AMI’s 1.5 μm, N-well, two polysilicon layers and two metal layers technology (Figure 8). Test structures for the evaluation of FGMOSFET’s were included, as well as a simple temperature controller circuit. Other isolated micro-heater structures were included for TCR measurements and anisotropic etching process optimization using TMAH.

**Figure 9.**
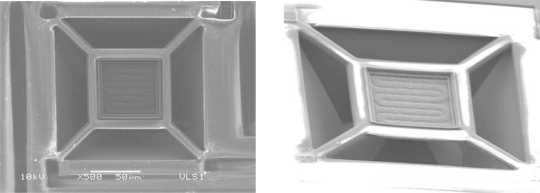
SEM microphotograph of the micromachined thin membrane with the micro-heater.

Figure 9 shows a SEM photo with some results of the micromachining of the micro-heater. As it can be seen, the anisotropic etching delivers a regular cavity with the shape of a truncated pyramid due to the (100) crystalline orientation of the wafer used in fabrication. The depth of the cavity is around 90 μm and is completed in about two hours of etching with TMAH. Other dimensions are specified in Figure 2. On the other hand, the geometry of the micro-heater should be optimized taking into account features as magnitude of resistance, area of the membrane used in the sensing area, and shape. An evaluation task of these parameters should be done to improve the performance of the heater considering issues as power consumption, technological parameters and the minimization of possible hot points along the polysilicon strip caused by localized current spots. Different shapes and resistance values can be designed, but they must fit to the design restrictions and must be based on minimum features allowed by the silicon foundry where the integrated circuit is going to be fabricated and the sheet resistance of the polysilicon.

## 3. Model for a FGMOSFET Sensor

Prior to the implementation of a transistor with the floating gate in direct contact with a deposited sensing layer, a simulation was outlined based in a FGMOSFET with the dimensions projected for a prototype of a gas sensor system. PSPICE is used to simulate the behavior of this device, with the assumption that a potential is present at the floating gate, as can be predicted from Equations (3) and (4). We should stress that since this potential is unknown until practical measurements can be done, in this simulation the magnitudes of the possible induced voltages are arbitrarily suggested to obtain a graph where the behavior of the structure *floating gate*/*sensing layer* can be predicted. An appropriate model for the FGMOSFET is used, where the dimensions of the channel width and length, and the coupling capacitance between control gate (Poly2) and floating gate (Poly1) are declared [32].

**Figure 10.**
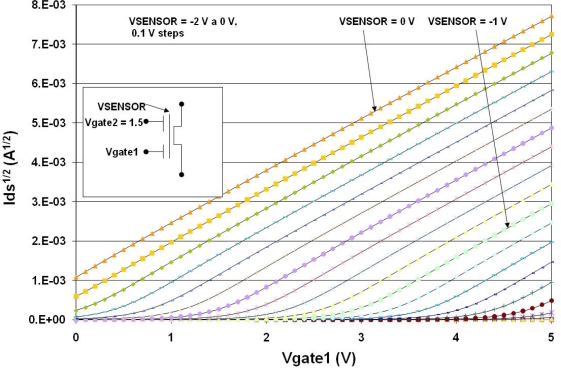
FGMOSFET with two control gates. Simulation considering a voltage variation on the floating gate. Vgate1 sweep: 0–5 V; Vgate2 = 1.5 V; Vsensor steps: 0–−2 V.

Initially, the behavior of a FGMOSFET with two control gates was simulated to obtain the I-V characteristics when a variable charge is present on the floating gate. Figure 10 shows a plot of Id^1/2^ *vs* Vgate1, with Vgate2 = 1.5 V. The parameter is Vsensor as the voltage due to the charge derived from the chemical reaction, ranging from 0V to −2 V with 0.1 V steps.

**Figure 11.**
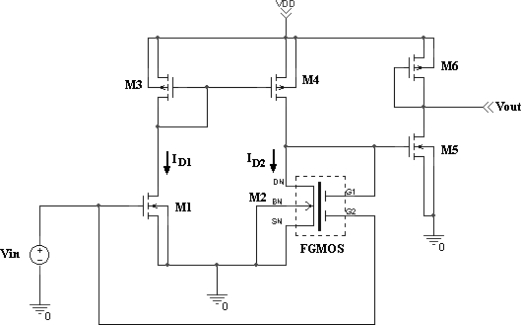
Two stage voltage amplifier with a differential input using a FGMOSFET.

It is shown that when the voltage present at the floating gate is modified, the transconductance curve of the FGMOSFET is shifted accordingly. Therefore at first instance, the device can respond to any charge present over the floating gate. Next, the FGMOSFET is included in a simple amplifier configuration, as the one shown in Figure 11. This is a two stage voltage amplifier with a differential input having one FGMOSFET as part of it. Vin is a biasing power supply to adjust the operating point of the amplifier to attain the highest gain. Thus, a simulation can be done considering an arbitrary variable voltage Vsensor, to evaluate the performance of such a circuit performing a possible cycle with different gas concentrations.

**Figure 12.**
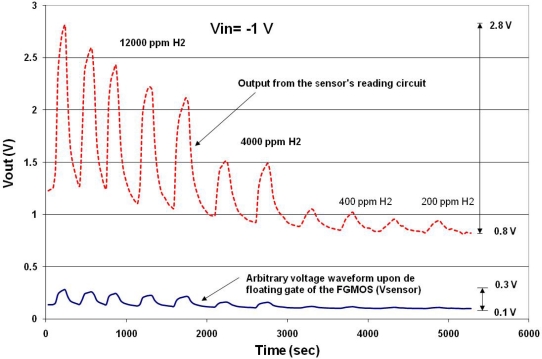
Arbitrary floating gate voltage varying in time, simulating a gas monitoring cycle, with supposed different gas concentrations.

To simulate the performance of the amplifier proposed in Figure 11, consider an arbitrary voltage variation in time for Vsensor like that shown in Figure 12. Suppose that the floating gate induced voltage has a DC level with a minimum voltage of 0.1V and a maximum level of 0.3 V representing also a hypothetical gas monitoring cycle from 12,000 ppm down to 200 ppm of hydrogen, for instance. Under these conditions, the bias operating point of the amplifier has to be defined for proper operation of the circuit.

**Figure 13.**
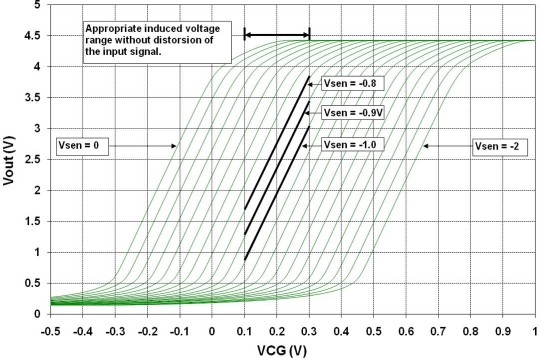
Transfer curve of the amplifier illustrated in Figure 11, with different bias voltages applied to Vin.

Therefore, a simulation is needed to determine the transfer curve that shows the highest gain and the voltage region for a non-distorted output signal. The corresponding simulation results are shown in Figure 13. It can be seen from this figure that the variable voltage signal upon the floating gate will be properly amplified if Vsen is between −0.8 V and −1.0 V. Otherwise, the output signal will be distorted and attenuated if other voltages are applied to Vsen. In practice, this should be a tuning operation for the amplifier to get a better signal reading. The result of the amplification with Vsen = −1.0 V is shown in Figure 12, where the voltage on the floating gate was amplified approximately 7.5 times with this simple amplifier configuration. These biasing conditions were particular for the proposed voltage (charge) variation shown in Figure 12 and they can be modified if different magnitude and offset values are present on the floating gate. This choice of Vin can be considered an advantage when using the FGMOSFET as the transducer, since tuning can be done depending on the gas concentration.

### 3.1. FGMOSFET Gas Sensor Model Validation

**Figure 14.**
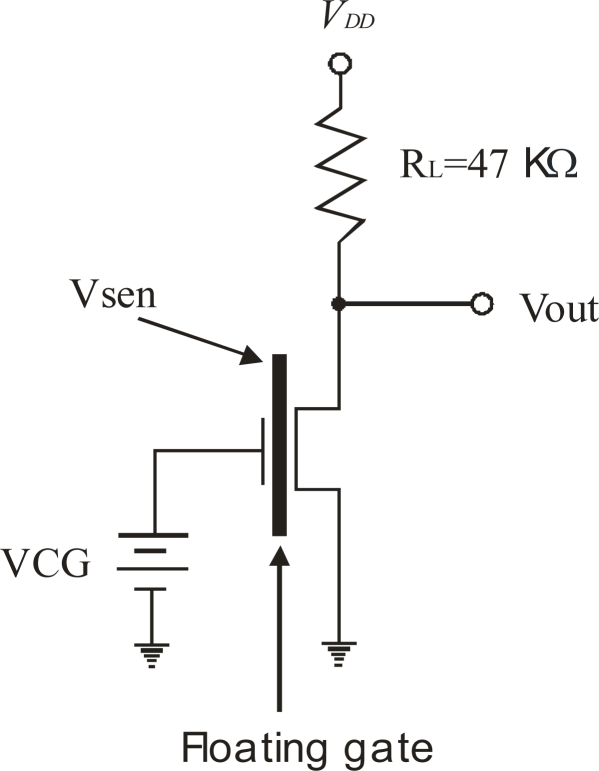
Simple amplifier used to simulate the performance of a FGMOSFET, with charge on the floating gate due to a chemical reaction.

Next, as simulations suggested that a FGMOSFET can reliably function as a gas sensor, an additional experiment was designed to observe such a feature. The experiment consists of using a conventional MOSFET in a very simple amplifier circuit. Figure 14 shows the basic amplifier for this experiment. Before the experimental measurement is performed, a previous simulation based on the model just described was done. From the simulation the plot for the output voltage (Vout) vs control gate voltage (VCG), with the voltage (charge exchange induced) due to the chemical reaction (Vsen) as a parameter, can be obtained.

Having in mind that one of the main objectives for portable gas sensor systems is low power consumption, a bias voltage *V_DD_* = 1.5 V was chosen. The amplifier used for simulations (Figure 14) has a load resistance of 47 KΩ that gives a good voltage gain; a voltage sweep for VCG from 0 V to 1.5 V is used as the independent variable and finally, the parameter Vsen varies from −0.3 V to 0.3 V, with 0.1 V steps.

**Figure 15.**
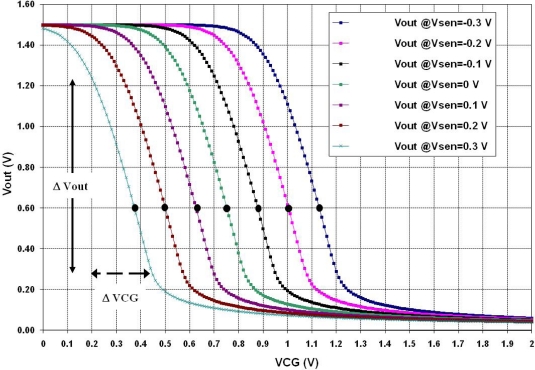
Transfer function of the voltage amplifier in Figure 14.

Figure 15 shows the simulated transfer function obtained with the voltage amplifier shown in Figure 14. The figure inset shows the different values of Vsen, where both negative and positive voltages where explored, since the polarity of the potential produced by the chemical reaction is also unknown. Thus, from Figure 15 it can be seen that the curve shifts to the left of the equilibrium signal (Vsen = 0, no chemical reaction) for positive Vsen voltages, otherwise the shift is to the right. From these curves, it is important to point out that they are also useful to choose the operating point of the amplifier so a reliable output voltage can be read, as mentioned before. For example, suppose that a Vsen = 0.3 V is induced upon the floating gate (see Figure 15), then in order to use the high gain region of the transfer curve, the VCG applied should be varied approximately between 0.2 V and 0.45 V, since in this range the FGMOSFET is in saturation. This range gives values for Vout between 1.24 V and 0.27 V, respectively. But if VCG is lower than 0.2 or higher that 0.45 V, low gains will result from the amplifier and the device will not be operating in saturation. A simulation for a DC analysis is enough to know the behavior of the system, since in practical situations the amount of the gas reacting with the layer is not expected to vary too quickly with time.

These results illustrate how the transfer curve of the amplifier is shifted as a consequence of changing the floating gate voltage. This voltage change can be due to a charge exchange between a thin sensitive film and a gas of interest in a convenient circuit structure.

### 3.2. Experimental Results (Model Evaluation)

**Figure 16.**
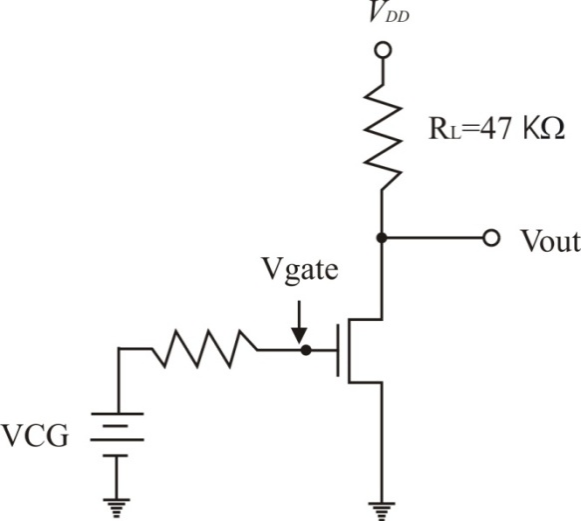
Experimental setup circuit for measuring propane, using a conventional MOSFET and a Fe_2_O_3_ sensing layer.

Since the experimental prototype shown in Figure 8 is not already finished, the operating principle can be proved alternatively using a conventional MOSFET. The experimental circuit used to validate the proposal is shown in Figure 16.

A conventional MOSFET transistor with a 47 kΩ load resistance and a bias voltage of *V_DD_* = 5 V. was used It is important to notice that the resistance connected in series with VCG represents the sensing layer. It consists of a separate glass substrate with a gas sensing layer, in series with VCG connected to the gate of the conventional MOS transistor. The experimental setup consists of a load resistance, a discrete MOS transistor, a sensing layer (1 cm × 1 cm) with interdigitated gold contacts, a variable DC power supply and a DC biasing power supply. Since the gate resistance of the MOSFET is very high. There are no leakage currents and allows for a correct measurement of the voltage present in the sensing layer when the chemical reaction is taking place.

**Figure 17.**
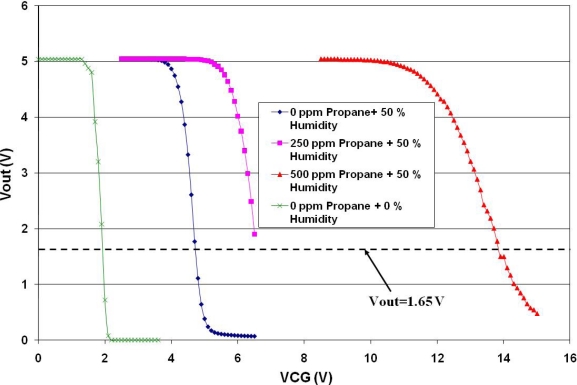
Experimental results measuring different concentrations of propane, using the experimental setup shown in Figure 16.

For this experiment, a layer of Fe_2_O_3_ (hematite) mixed with pyrrole was used to measure the electrochemical potential created by the chemical reaction with propane in the presence of 50% of humidity in the experimental chamber, keeping the layer at 30 °C. The layer was deposited by spray pyrolisis upon a glass substrate and then interdigitated gold contacts on about a 1 cm × 1 cm area were evaporated. The results are presented in Figure 17. The transfer curve with Vsen = 0 V has its high gain region between 1.7 V and 2 V of VCG. Good resolution in the voltage readings was obtained for propane concentrations of 250 ppm and 500 ppm. Based on Figure 14, an equivalence can be derived for the amplifier in Figure 16. The voltage drop on the sensing layer is given by the difference between the voltage read at the MOSFET gate and the VCG voltage, as follows:

(6) Vsen=Vgate−VCG

where Vgate is the gate voltage of the MOSFET measured with reference to ground. Since the output voltage, Vout, depends on the amplifier’s gain, it is advisable to take the Vgate reading using a circuit operating point that delivers the highest gain. For the case illustrated in Figure 17, this value corresponds to Vout = 1.65 V. Table I shows the electrochemical potential produced by the chemical reaction between the hematite film and propane.

As can be seen in Table 1, easy reading of voltages was accomplished after the chemical reaction of the hematite with propane. Good resolution was obtained with values of 0 ppm, 250 ppm and 500 ppm of propane, suggesting that interesting work can be done in designing a whole system that can integrate the sensor with the electronics, based in FGMOSFETs with the alternative of sensing a gas through the shift of the threshold voltage instead of the layer resistance variation, as is used in most of the gas sensors reported [6,8,14,33–36]. For the amplifier using the conventional MOSFET, measuring Vgate and using Equation (6), is enough to deduce the electrochemical potential, but it is important to say that the last experiment cannot be done if an FGMOSFET is used, since there is no way to directly read the potential of the floating gate. Therefore, an indirect method must be used to achieve this. The next section shows the way this can be achieved. It was previously demonstrated [37,38] that metal oxide thin films used as gas sensitive layers can be purged so they can recover the initial resistance value. This can be done by applying an appropriate thermal treatment. For instance, in [37] they use a pulse operating mode for the micro-heater. During measurement, the film is heated up to 200 °C during 8 seconds in presence of the gas. Then, to reset the initial condition, it is heated up to 585 °C during 5 seconds in clean air, resulting in a reversible reaction.

### 3.3. Extrapolation to a Gas Sensor with an FGMOSFET

Based on Figure 15, it can easily be shown that:

(7) Vout=G×VCG+Vsen

where G is the gain of the amplifier. Once again, using the operating point where the amplifier has its higher gain and reading its corresponding Vout, then Vsen can be determined, as well, for a sensor structure based on an FGMOSFET. From Figure 15: Gmax = 0.8, Vout @ Gmax = 0.6 V. Then, reading the operating point for each curve in the graph, effectively results in the proposed voltages in the floating gate, proving that Equation (7) and the method used, is very reliable. Table 2 shows the calculations for the six curves illustrated in Figure 15, where the floating gate potentials used are obtained after using Equation (7).

Based on the above analyses and results, a similar behavior can be observed between the simulated and experimental transfer curves suggesting that a voltammetric sensor can be designed by using an FGMOSFET as the transducer. Although in this work a thin film not deposited over the floating gate of the MOS transistor was used, the similarity of simulation with the experimental results illustrates the possibility of finding the same behavior once a metal oxide film is deposited over the floating gate of the integrated system. Work should be done forward to prove that the mentioned chemical injection also works as the experimental results suggest.

## 4. Conclusions

With the proposal discussed in this work, it is demonstrated that a standard CMOS technology can be compatible with micromachining processes used for MEMS structures, using a post-processing etching step for micromachining a membrane that contains the micro-heater. The design must allow for evenly heating the layer up to 400 °C and to take advantage of the cavity created for thermal isolation purposes. This point is very important for isolating the gas sensor’s temperature from the substrate containing the signal processing electronics, making possible a monolithically integrated system. Besides, it was demonstrated that a FGMOSFET is a very reliable device that can be used for gas monitoring. The design is based on a two polysilicon and two metal layers CMOS technology. A model was proposed for simulation purposes taking into account the electrochemical potential derived from a chemical reaction. This is true also with sensing layers that operate even at low temperatures, such as 30 °C, like Fe_2_O_3_ or polymers working at room temperature. A model is proposed from where a gas sensor using a FGMOSFET can be designed. This model was validated through a conventional MOSFET and using a layer of Fe_2_O_3_ connected in series with the transistor and performing a measurement of propane. Although some gas sensors have been proposed in the past, this is a different approach that can be configured for organic or inorganic sensing.

## Figures and Tables

**Table 1.** Voltages in the Fe_2_O_3_ sensing layer (Vsen), due to the chemical reaction with propane, using the circuit of Figure 16.

Concentration of Propane	Vgate (V)	VCG (V)	Vsen (V) @ Vout = 1.65 V
0 ppm	1.976	1.976	0
0 ppm + 50 % humidity	1.976	4.8	−2.824
250 ppm + 50 % humidity	1.97	6.3	−4.33
500 ppm + 50 % humidity	1.97	12.5	−10.53

**Table 2.** Calculation of Vsen from Figure 15, using Equation (7) with Gmax = 0.8.

**Vout (V)**	**VCG (V) @ Gmax**	**Vsen (V)**
0.6	0.625	0.1
0.6	0.5	0.2
0.6	0.373	0.3
0.6	0.876	−0.1
0.6	1.0039	−0.2
0.6	1.13	−0.3
